# An Augmented Reality Periscope for Submarines with Extended Visual Classification

**DOI:** 10.3390/s21227624

**Published:** 2021-11-17

**Authors:** André Breitinger, Esteban Clua, Leandro A. F. Fernandes

**Affiliations:** Instituto de Computação, Universidade Federal Fluminense (UFF), Av. Gal. Milton Tavares de Souza, Niterói 24210-346, RJ, Brazil; esteban@ic.uff.br (E.C.); laffernandes@ic.uff.br (L.A.F.F.)

**Keywords:** computer vision, deep learning, mixed reality, object detection, periscope, synthetic data, submarine, transfer learning

## Abstract

Submarines are considered extremely strategic for any naval army due to their stealth capability. Periscopes are crucial sensors for these vessels, and emerging to the surface or periscope depth is required to identify visual contacts through this device. This maneuver has many procedures and usually has to be fast and agile to avoid exposure. This paper presents and implements a novel architecture for real submarine periscopes developed for future Brazilian naval fleet operations. Our system consists of a probe that is connected to the craft and carries a 360 camera. We project and take the images inside the vessel using traditional VR/XR devices. We also propose and implement an efficient computer vision-based MR technique to estimate and display detected vessels effectively and precisely. The vessel detection model is trained using synthetic images. So, we built and made available a dataset composed of 99,000 images. Finally, we also estimate distances of the classified elements, showing all the information in an AR-based interface. Although the probe is wired-connected, it allows for the vessel to stand in deep positions, reducing its exposure and introducing a new way for submarine maneuvers and operations. We validate our proposal through a user experience experiment using 19 experts in periscope operations.

## 1. Introduction

Submarines are among the most capable and strategic naval units to operate in areas where the enemy exercises some degree of control. The procedure adopted by many countries suggests that submarine actions are the priority in enemy monitoring, not only for reducing the control exercised by them but also for supporting other forces’ actions. The availability and presence of submarines also significantly increase dissuasion potential due to the uncertainty of its actual position [1].

**Figure 1:**
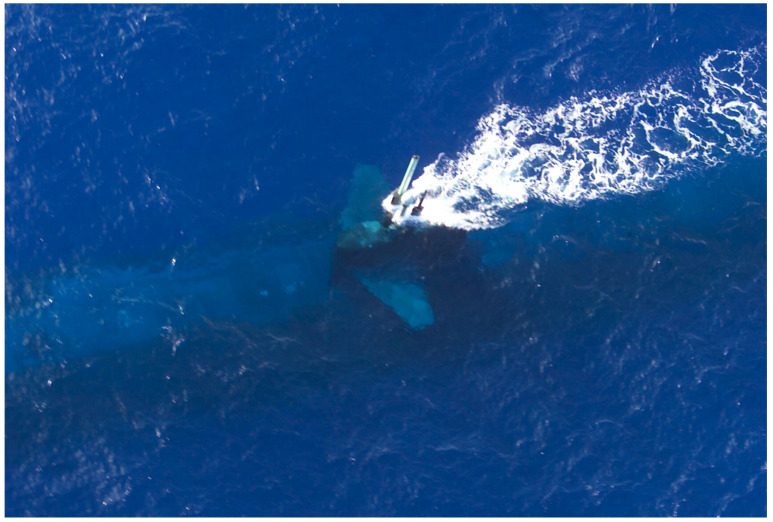
Periscope exposure at periscope depth. Source: [2].

One critical maneuver for submarines is the *periscope observation*, which requires the ship to navigate at periscope depth (Figure 1). This exposition is strategically dangerous because the submarine can be detected by nearby enemies visually or by radar, becoming vulnerable. The periscope observation is made using a long periscope, a piece of optical equipment capable of rotating $360^{\circ }$, giving a panoramic view of the surface. Due to the degree of danger, this exposure should occur for just a few seconds. It must also be performed by a trained officer operating the periscope, which is assigned to identify contacts in the visual range considered potential hazards during that short period.

Submarine Discretion Fee (SDF) is defined as a percentage ratio between the sum of the indiscretion periods (mast exposed) and the total submarine operation time. The objective of the submarine commander is to accomplish his mission while obtaining the minimum possible SDF.

The Brazilian navy adopts the technique called “perisher”, which was developed by the British royal navy [3]. This technique maximizes the amount of information obtained from the periscope while minimizing exposure. Intermittent exposure reduces radar and visual detection probability. In the “perisher,” the action of performing a horizon scan takes 30 s, only to check whether there is any hazard at the field of view, without any further observation of the detected contacts. A posterior investigation of each contact is made for 20 s on each one for identifying the elements. The goal is to estimate the bow angle, measure the distance to the contact with a stadiometer, and calculate the observation interval. Such a calculation is based on the contact’s distance and its maximum speed. Usually, the periscope officer mentally calculates the maximum amount of time to observe the contact, again putting the submarine at risk.

Deep Neural Networks (DNNs) have shown significant improvements in several application domains, including Image and Signal Processing. In Computer Vision, a specific type of DNN, known as Convolutional Neural Networks (CNNs), has revolutionized the state of the art of object detection and recognition, achieving faster and more accurate results [4].

This paper proposes a Mixed Reality (MR) periscope device, which is a novel and powerful solution capable of decreasing the periscope’s exposure time and drastically increasing the observation tasks through Computer Vision techniques. Our solution is based on a wired probe that carries a high-resolution $360^{\circ }$ camera and is connected to a commercial Head-Mounted Display (HMD) device. We use different Computer Vision and Deep Learning techniques for surface elements’ classification and distance inference, which have the potential to dismiss the use of conventional stadiometer requirements. We also propose including different navigation information at the HMD display using Augmented Reality (AR) strategies.

We believe that our proposal will introduce a new way of operating periscopes and performing submarines operations in the near future. The Brazilian Navy Research Center (CASNAV) and the Submarine Defense Division have sponsored this work. Our main contributions can be summarized as:

- A new architecture for submarines periscope using Extended Reality (XR) devices and approaches;
- A training strategy using synthetic images to fine-tune a YOLO-based solution for ship detection and classification in pictures taken from a periscope point of view;
- A ship distance estimation solution for recognized ships;
- An open dataset composed of 99,000 synthetic images of five (strategic) classes of ships; and
- A user-experience experiment that validates the usage of Virtual Reality/Extended Reality (VR/XR) devices for periscope operations.

The paper is organized as follows: Section 2 presents the correlating fields of knowledge that inspired our work and discusses solutions based on synthetic images for the training classification models. Section 3 summarizes our proposal, presenting our novel periscope architecture, our proposed training strategy for the detection and classification model, and our object distance estimation approach. Section 4 and Section 5 present our experimental evaluation, associated studies, analysis, and a user experience test with submarine officers. Finally, Section 6 concludes our work and discusses the future possibilities of our proposal.

## 2. Related Work

Stanton et al. [5] present all challenges, risks, and strategic solutions related to submarine operations. Our work is inspired by the related issues raised in the document, where it is shown that standing at sea level breaks the submarine’s invisibility and makes it vulnerable to other vessels and air vehicles. Stanton et al. [5] also explains why the transition from deep to periscope depth is one of the most dangerous operations due to the potential to collide with surface vessels.

Although we believe our work is the first to introduce a $360^{\circ }$ camera and AR based periscope, other works take advantage of the combination of these technologies for surveillance and security. Grabowski [6] presents the requirements and the capabilities of piloting and navigation with immersive technology on safe and effective marine transportation using Wearable and Immersive Augmented Reality (WIAR). The authors establish the link between technology decision support and improved maritime safety, facing the problems inherent in technology introduction in marine transportation.

Laera et al. [7] presents a systematic review analyzing the publication type, the AR device, which information elements are visualized and how the information is displayed, based on the information, we displayed on the XR device basic information, like own ship speed and relative heading.

According to Milgram et al. [8], the Reality-Virtuality Continuum is constituted by different levels of immersion, going from the Real Environment, Augmented Reality, Augmented Virtuality and Virtual Reality. While our solution resides at the Augmented Reality stage, we use Augmented Virtuality devices as interfaces.

The International Regulations for Preventing Collisions at Sea (COLREGs) defines several rules to prevent collisions [9]. Collision is particularly dangerous to the submarine because there is a significant probability that other ships are not aware of the submarine’s position. Therefore, an XR solution with a visual camera can recognize dangerous elements. The literature on XR periscope or Computer Vision applied for submarines’ periscope is almost nonexistent. Still, this problem faces similar issues with detecting cars or traffic signs using a camera in autonomous or semi-autonomous vehicles. De Mello et al. [10] has proposed a method to generate artificial traffic-related training data for deep traffic light detectors, offering a solution using deep neural networks for problems associated with autonomous driving. Concerning vessel detection and classification, Kim et al. [11] proposed a novel probabilistic ship detection and classification system based on deep learning using a dataset of images available on the web. However, the annotation data from different classes of ships are not vast and not trivial to be solved. Lee et al. [12] Presented an Image-based ship detection using deep learning, it uses a CNN to detect objects and then classify as ship, speedboat, and buoy.

In this work, we intend to detect vessels in images using Computer Vision techniques. To create a dataset, we used the Bridge Navy Simulator for producing a set of renderings (i.e., synthetic images) of strategic ship classes for submarines operations. Ward et al. [13] proposed a synthetic dataset to classify ships from satellite images. Instead, we took a similar approach but developed a dataset composed of synthetic images with a different camera position, constrained to the submarine periscope point of view.

As discussed in [14], transfer learning techniques reduce the need for large datasets due to the generalization ability of the parameters learned by the lower layers of the CNN from public datasets, like MS COCO [15]. We used a pre-trained YOLOv4 [16] CNN to get such parameters and train the weights of the classification layers with our synthetic dataset.

## 3. The XR Periscope

We propose a novel generation of submarine periscopes based on a high resolution $360^{\circ }$ camera mounted in a floatable probe, coupled to a Mixed Reality HMD device. The probe is projected in such a way that it can be dragged by an underwater vehicle (submarine). It has a precise hydrodynamic to achieve stability in the camera image and enough height to extend the horizon line and detect surface elements and vessels. The $360^{\circ }$ video is streamed to the HMD device, placed inside the submarine. The movement of the HMD performs the selection of the $360^{\circ }$ video area being viewed by the periscope operator and processed by the Computer Vision module. AR features are inserted in the image, including vessel type, bearing, and distance calculation information.

**Figure 2:**
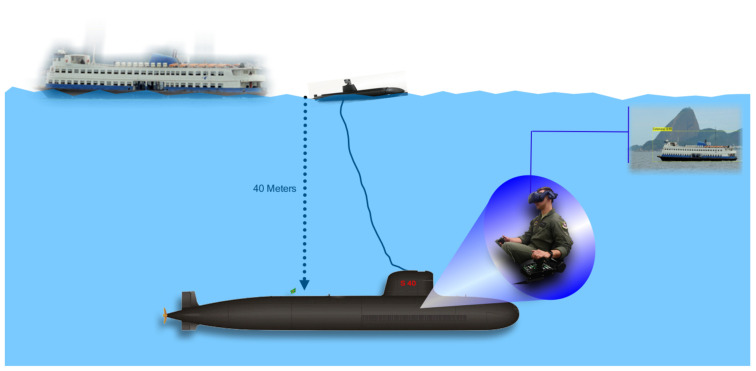
Overview of the proposed solution.

**Figure 3:**
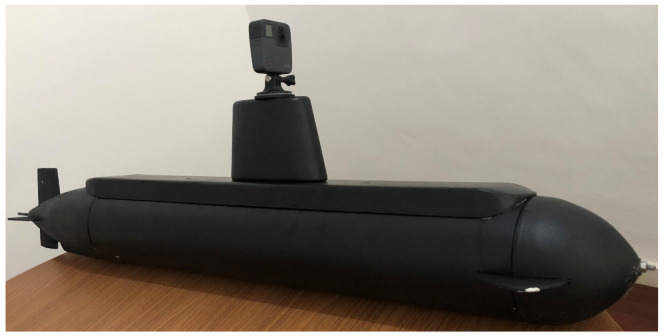
Our developed probe carrier mockup with a $360^{\circ }$ camera mounted at the top of it.

The submarine velocity at deep waters is around 5 knots. In this sense, the probe was developed so that it has stability and hydrodynamics at this speed. In order to avoid wave and water drops interference in the images, the camera was projected to be mounted at 40 cm above sea level. The camera is attached to a protected HMDI cable that connects the devices with the submarine. Figure 2 shows the schematic view of our solution, and Figure 3 shows our operational-developed probe, with the camera mounted at the top.

The targeted areas of interest are processed by deep learning algorithms using a CNN for object detection and classification (Section 3.1). The CNN is trained with a dataset composed of 99,000 synthetic images (Section 3.2) of ships generated using the Brazilian Navy Bridge Simulator from the Naval Systems Analysis Center (CASNAV). Our dataset is open access and collaborative. Although we have built our system for the set of vessels considered most important for the Brazilian submarine operations, expanding and including more vessel models in this dataset is straightforward.

The distance to each target is estimated by the relationship between the actual known height of the detected and classified vessels and the vessel’s size in the recorded image (Section 3.3).

### 3.1. Detection and Classification Stage

Once the images are transmitted to the submarine, we apply different Computer Vision approaches for enhancing and detecting contacts above the sea. We have used YOLOv4 [16] in the current implementation of our system, but one can also apply other object detection and classification solutions. The idea behind YOLO is that a single neural network is applied to an entire image. This allows YOLO to reason globally about the image when generating predictions. The YOLO network divides the image into a S×S grid of cells, where *S* is a hyperparameter defined by the user according to his needs and the characteristics of the input dataset. YOLO predicts *B* bounding boxes for detected objects in each grid cell and computes *C* class probabilities for those objects. The number of classes *C* depends on the dataset, while the user provides the hyperparameter *S*.

We used a fine-tuning strategy to train our model. So, we took the YOLOv4 trained on the MS COCO dataset [15] and specialized its training on our new synthetic dataset. The first few convolutional layers learn low-level features (curves, color, edges, blobs). As we progress through the network, it learns more mid/high-level features or patterns. We freeze these low-level features trained on the MS COCO and only retrain high-level features needed for our new image classification problem, replacing the classification layer with our setting, with a different number of classes.

After the training stage, the CNN returns several axis-aligned bounding boxes whenever we send an input image. Each bounding box is defined by (x,y), *w*, and *h*, where (x,y) is the center of the box, and *w* and *h* are its width and height, respectively. By multiplying the conditional class probability and the individual box confidence predictions, we get the class-specific confidence score for each box and use this data to draw the boxes on the output image. We use the height of the box and additional information about the ship’s class to calculate the object distance to the probe.

### 3.2. Training Data

**Figure 4:**
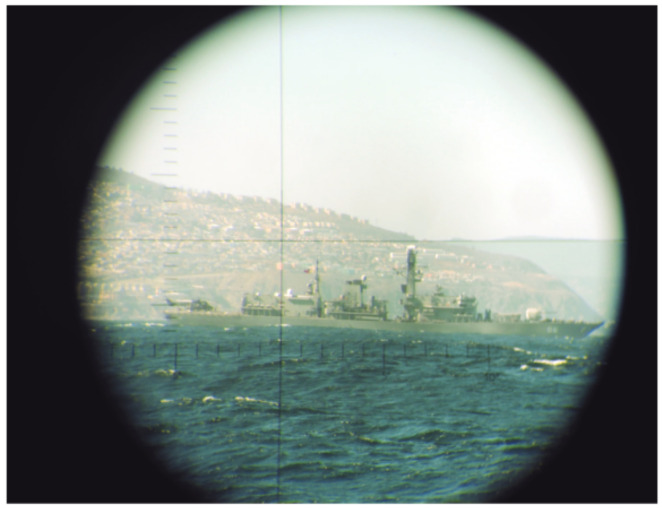
Periscope point of view. Source: adapted from [17].

**Figure 5:**
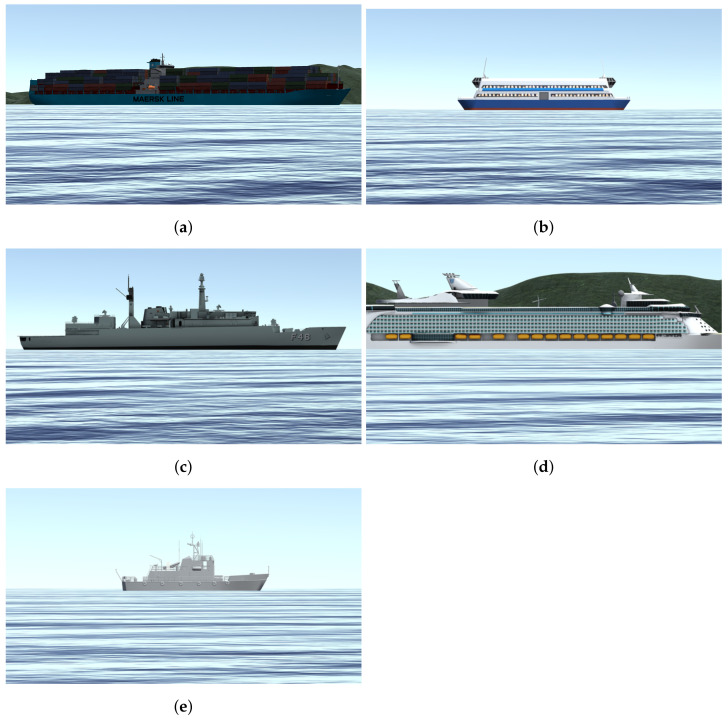
Classes of vessels considered while training the model using synthetic data; (**a**) Container Ship; (**b**) Ferry; (**c**) Frigate; (**d**) Passenger Ship; (**e**) Yard Ship.

Due to the periscope’s positioning and our $360^{\circ }$ camera elevation above water, it is plausible to state that the objects on the surface necessarily cross the horizon line. We generated our training data with this concept in mind and placed the virtual camera used in the simulated scenario about 40 cm above the water level. Figure 4 illustrates this point of view and configuration, and Figure 5 shows a set of samples of synthetic images generated using the CASNAV Bridge Simulator for the dataset. Table 1 summarizes the distribution of images in our dataset, according to the distance and presence or absence of background.

We developed an application to extract synthetic images of five classes of ships implemented by the CASNAV Bridge Simulation System. We generated one image for each combination of ship class and degree step in a bow angle (from $0^{\circ }$ to $359^{\circ }$). This set was combined with different backgrounds and distance conditions, as described in Table 1, leading to 3960 raw images per class and 19,800 images in total. For each “closest” positioning distance, we generated the image with and without background, so our CNN network could learn to detect small details available at each vessel. Image without background means that besides the ship, the image also contains water and sky (e.g., Figure 5b,c,e). An image with a background means that it also has land behind the vessel (e.g., Figure 5a,d).

The artificial dataset, including only those 19,800 images mentioned above, is quite repetitive since we assume $1^{\circ }$ steps in bow angle and too clean renderings (i.e., without noise). As a consequence, in our first experiments, we noticed that our results presented significant overfitting rates. To avoid this, we included in our dataset new images generated through data augmentation strategies. We found that the following types of data augmentation were the most relevant for training the detection and classification model: Gaussian noise, impulsive noise, blur, shadow, shear, and small rotations restricted to angles that can be included by sea waves’ movement. The proportion of the vessels that are being classified, compared with its inclination due to waves movements are almost insignificant. For this reason, we did not included the tilt rotation. However, in future works, where we intend to include more types of ships, this feature must be considered.

**Figure 6:**
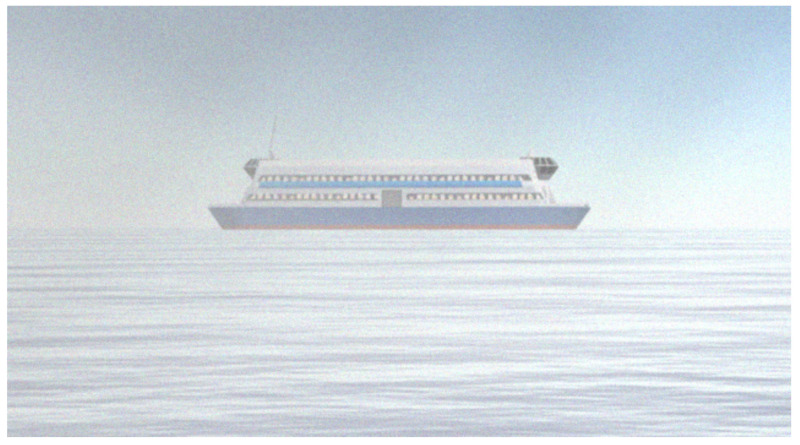
Example of an augmented synthetic image used for training the classification module. This image includes Gaussian noise and blur.

We generated four augmented images for each synthetic image in the initial collection of renderings, assuming random values defined between a minimum and a maximum parameter for each original image. By doing so, we end up with a synthetic image dataset composed of 99,000 images. Figure 6 shows an example of an augmented synthetic image.

In order to fine-tune our CNN model, it is necessary to have all the data with precise annotation. Due to the large number of images, it was impossible to label them one by one manually, so we implemented an algorithm to tag them in a semi-automatic fashion. The script was developed in AutoIt [18] and after the user inputs the position of each ship in each distance at $90^{\circ }$, $60^{\circ }$, and $30^{\circ }$, it calculates and generates a file for each image in the YOLO’s annotation format:


class x y width height

### 3.3. Distance Estimation Stage

**Figure 7:**
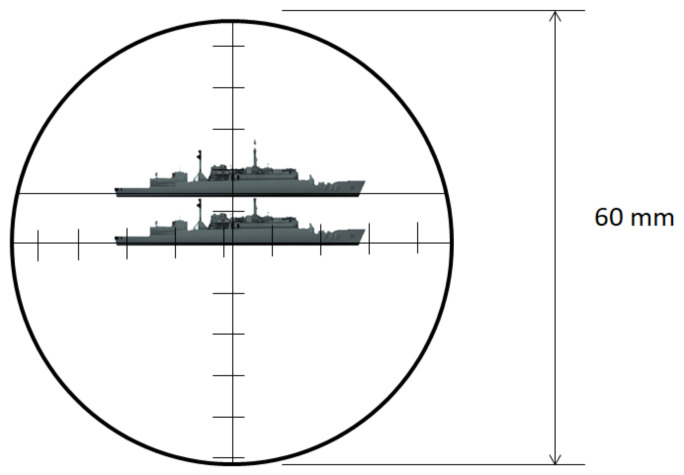
Typical stadiometer split image.

The optical periscope allows estimation of the distance of a known object by a stadimetric range finding method. It is a process based on triangulation in which the angle subtended by a target of known height (usually from the waterline to masthead height) is measured by vertically displacing the fields of view in each half of a split lens. This optically measured angle and the operator-inserted target height are used to estimate the distance to the target in yards (a.k.a. target range). A typical stadiometer split image can be seen in Figure 7.

The expression to manually compute the target range (TR) is:

(1) $$TR=\frac{TargetHeight\times FocalLength}{StadimeterSplit}.$$

**Figure 8:**
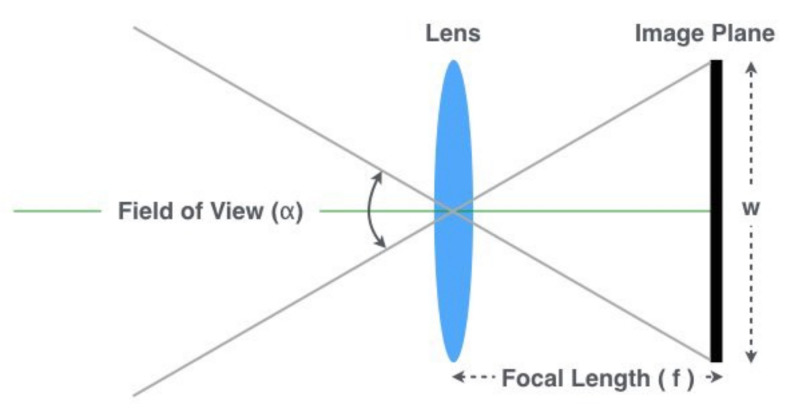
Camera geometry.

The distance from objects is the most critical calculation when the submarine is at periscope depth and detects a vessel. The faster and precise the distance is estimated, the less time the periscope has to be hoisted. We have developed a stadimeter-inspired method for estimating the distance of ships of available classes through the classified image results. Our approach is based on triangle similarity, where three parameters are necessary to calculate the ship’s distance:

1. The object’s height in the 3-dimensional space (*H*): Once we have classified the vessel, it is possible to retrieve its known height since this information is usually available to the periscope officer;
2. The object’s height in the image (*P*): After applying the detection and classification model, we get the axis-aligned bounding box of each detected ship and its respective confidence score indicating how good the detection is. We assume that the height of the bounding box is the height *P* of the object in image space, measured in pixels;
3. The focal length of the camera(*f*): It can be found in the camera’s specifications or estimated using one of the methods explained below. As depicted in Figure 8, it can be computed as:
  (2) $$f=\frac{W}{2}\times \cot\left(\frac{\alpha }{2}\right),$$
  where α is the field of view angle, and *W* is the image width. Our approach for computing the focal length is:
  (3) $$f=\frac{P\times D}{H},$$
  where *D* is a known distance of an object used for calibration, *P* is the height (in pixels) of the object in the image, and *H* is the known height of the object’s class in the 3-dimensional space.

As mentioned in Section 3.1, the bounding box of each detected object is defined by (x,y), *w*, and *h*, where (x,y) is the location of center of the bounding box, and *w* and *h* are its width and height, in pixels. As the heights of the trained classes are known, after the model returns a bounding box with an appropriate confidence level, it becomes possible to calculate the distance of a target object to the submarine using:

(4) $$D=\frac{f\times H}{P},$$

where P=h by construction.

## 4. Experiments and Results

**Figure 9:**
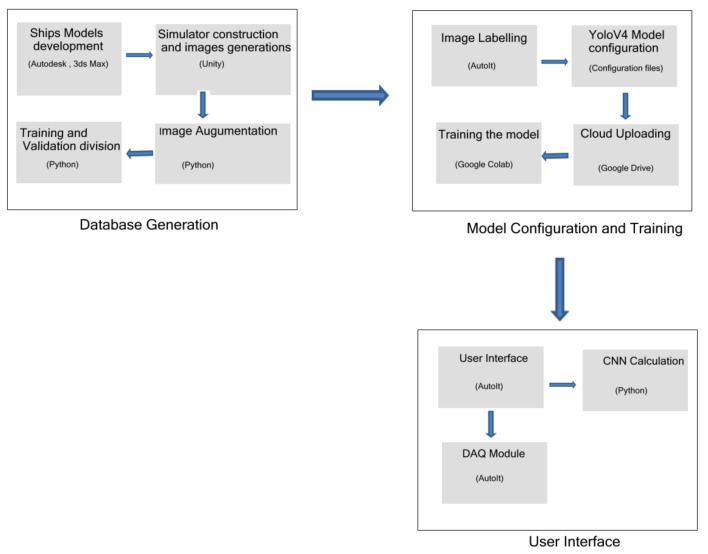
Steps Flow Chart.

Figure 9 shows a flow chart of the steps. The first stage is called Database Generation and is composed by the simulator data generation and acquisition, followed by the data augmentation, training and data set validation. The second stage is named as Model Configuration and Training and starts with the image labeling process through our developed system, the Yolo framework execution and training the data into the cloud environment. Finally, the third stage is the final user process, composed by the real time CNN classification and Data Acquisition for enhancing the classified data with new images.

The following subsections describe how we have implemented the proposed system, trained our detection and classification model, evaluated our results using synthetic and natural images, and performed tests simulating real conditions. The following steps summarize our solution:

1. Ship modeling: 3-Dimensional ship models were developed using Autodesk and 3DS Max;
2. Images generation: The images were extracted from our Bridge Simulator, which is built in Unity;
3. Dataset augmentation and split: A Python script was developed to perform an image augmentation and dataset split in training and testing subsets;
4. Image labelling: An AutoIt script was developed to label the images;
5. Model training: The configuration files and images were uploaded to the cloud for training the model on Google Colab Pro machines;
6. CNN application and user interface: The CNN classification is performed;
7. Data acquisition: The data acquisition (DAQ) module was developed in AutoIt.

### 4.1. Model Configuration and Training

As described in Section 3.2, the dataset was generated using synthetic data produced by the CASNAV Bridge Simulator, equally distributed in the five classes as can be seen in Table 1, and extended through data augmentation approaches. Before training the model, the dataset was randomly split in a *training* subset with 79,200 images and a *testing* subset composed of 19,800 images.

**Figure 10:**
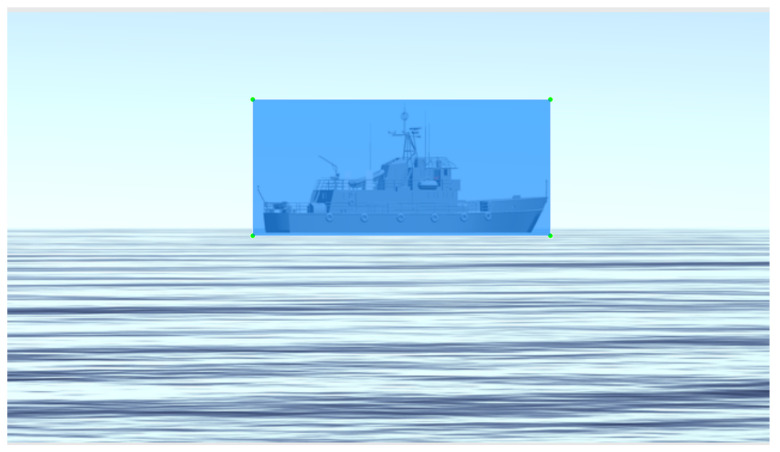
Labeled image. Blue means the box in which the object is contained.

To assist the dataset labeling process, we developed a program that semi-automatizes the task. Figure 10 shows an example of a labeled image produced by our program.

The model was trained using the publicly available Darknet, which is an open-source neural network framework written in C++ and CUDA. It includes the implementation of a consolidated state-of-the-art object detector, YOLOv4.

We have used default values for almost all YOLOv4 hyperparameters. The only exceptions are: the input image size, which was set to 640×352 pixels; the batch size was set to 64, and subdivision to 16; the size of the last convolutional filters before each of the YOLO layers was set to classes+5×3=30 as, according to Darknet documentation, it depends on the number of classes. The model was retrained using 70 K iterations, keeping the weights for every 10 K iterations. The number of steps was set to 56 K following the recommendation of 80% for the number of batches for this hyperparameter.

Using the augmented labeled set of synthetic images, we performed the fine-tuning strategy starting from the yolov4.conv.137 layer of a YOLOv4 model pre-trained on the COCO dataset. Using these weights helps the object detector be way more accurate and not train for too many epochs.

All training and interference processes were performed on Google Colab Pro [19], which is a research project for prototyping machine learning models on powerful hardware options such as GPUs and TPUs. It provides a serverless Jupyter notebook environment for interactive development. The configuration used to train our model was: Intel(R) Xeon(R) CPU @ 2.00GHz, 26GB RAM, 200GB Hard Drive and a NVIDIA Tesla V100-SXM2-16GB GPU.

### 4.2. Data Acquisition Interface

**Figure 11:**
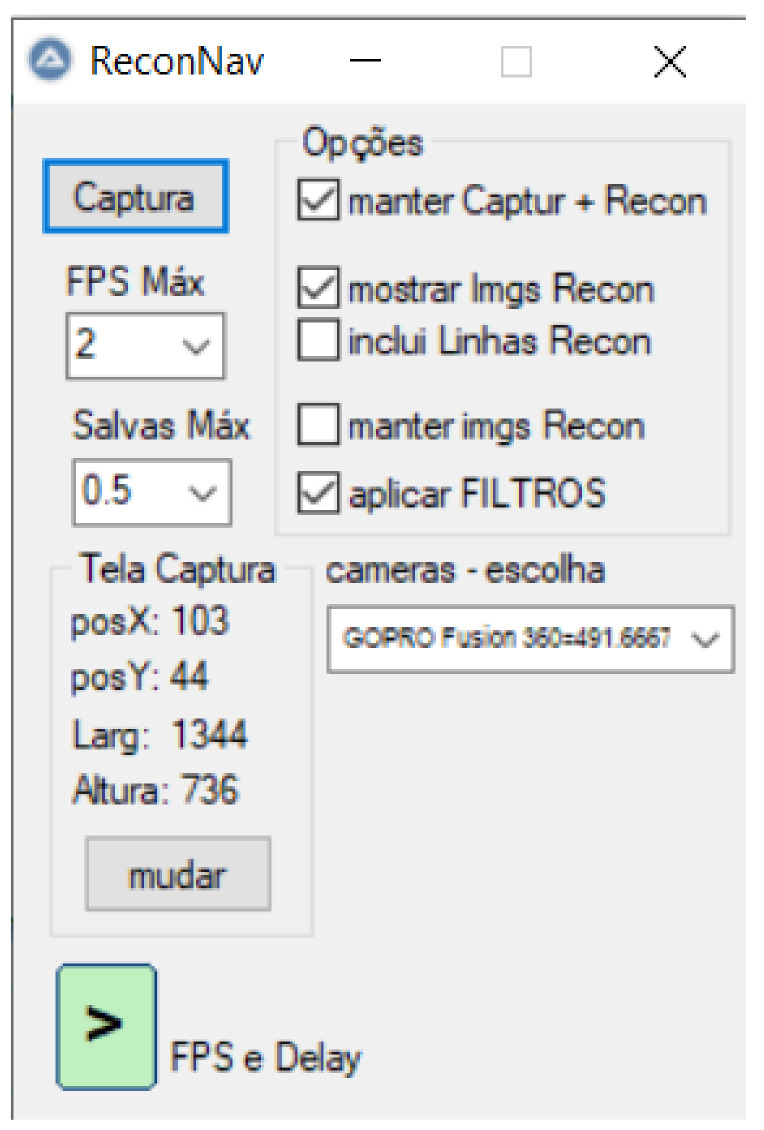
Tool for helping vessels classification. The interface is in Portuguese.

In order to improve the labeling process, we developed a dedicated tool, presented in Figure 11. The main objective of this tool is to allow the visualization of a video in real-time with recognition boxes around the objects defined at each frame. These boxes have on their top the type of recognized object (in this case, the ship type) and its distance in meters. The second function of this tool is to allow the images with the boxes in the saved objects to be stored for logging or event recording purposes. Finally, this program also intends to store the original images and their reconnaissance parameters so that users can include and train the model for new vessel types in the future.

The system saves the object type, position, length, height, and distance in an input format compatible with the YOLOv4 system to enhance model training.

### 4.3. Classification Results

The model was tested with the testing subset and with real vessel images, always restricted to the point of view of the periscope. Next, we describe each step in detail.

#### 4.3.1. Detection and Classification

**Figure 12:**
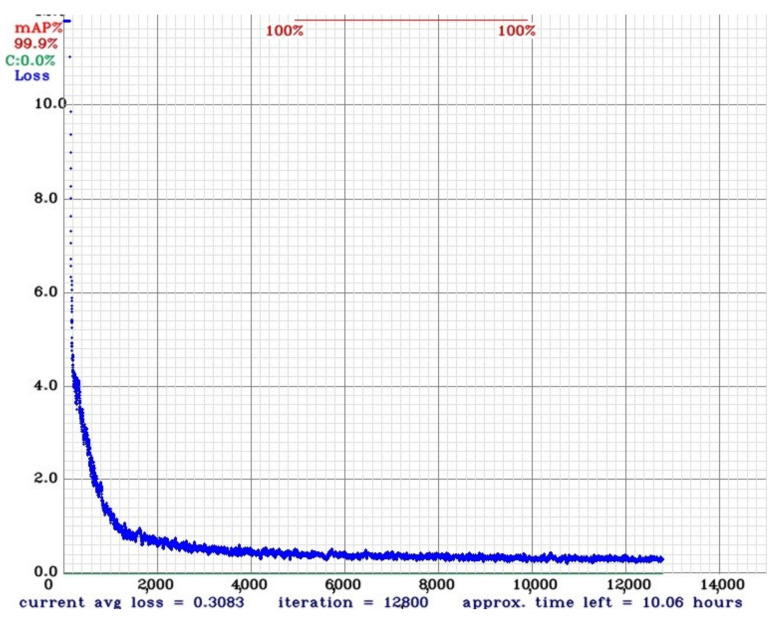
Average loss during the first 12 K iterations.

We used the Mean Average Precision (mAP) score to measure the quality of the results. After the model training, we noticed a fast convergence to an optimal average loss, as can be seen in Figure 12.

The mAP results stabilized when using 10 K iterations or more. After running the model with different weights on real data previously acquired, it was empirically defined that the best-achieved result was at 20 K iterations.

The precision results can be checked in Table 2. The global model results are: Precision =1.00, Recall =1.00, F1-Score =1.00, True Positives =19,787, False Positives =24, False Negatives =13, and Average IoU (Intersection over Union) =0.90.

**Figure 13:**
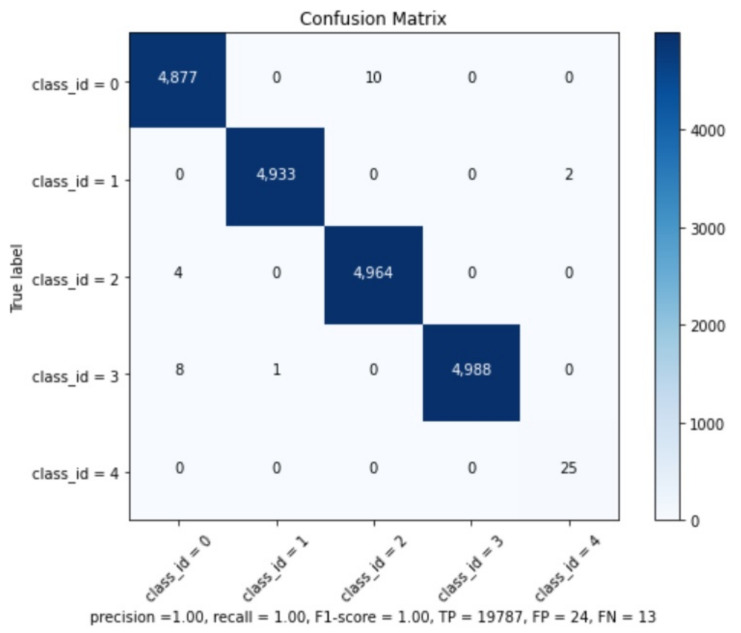
Confusion Matrix.

We believe that this good mAP result is related to the similarity of training and testing subsets and because the best weights were collected after 20 K interactions. It is well-known that with the increase of iterations, the model might present overfitting [20]. However, it is important to remember that the operational CNN will be applied on real images and not on synthetic ones, which are very different from the training subset and, as such, not prone to this overfitting issue. The confusion matrix can be seen at Figure 13 and confirms the information from Map analysis.

**Figure 14:**
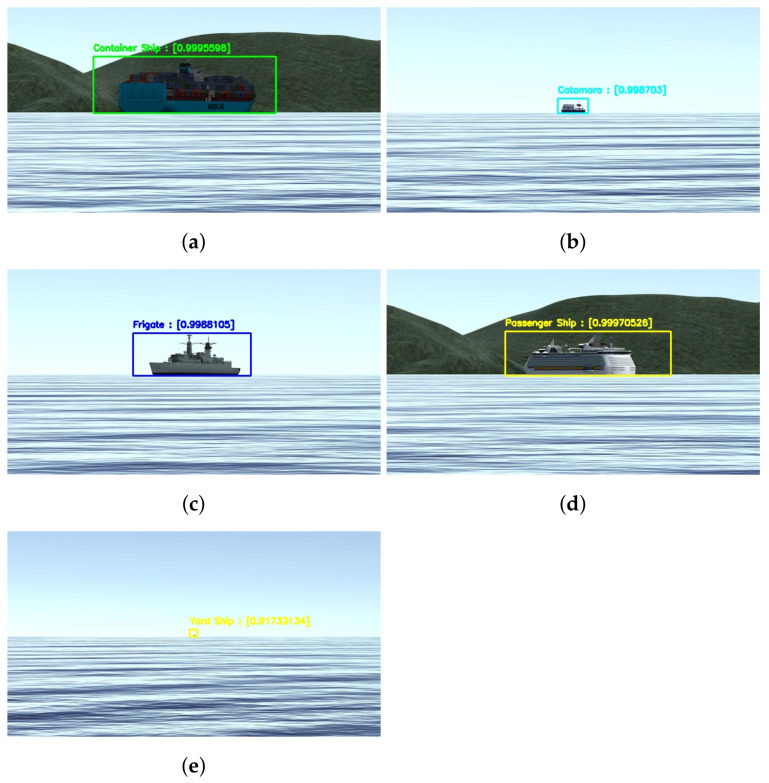
Some results from the detection and classification model applied on synthetic images. The distance is estimated in meters, and the bow angle is given in degrees. (**a**) Container Ship at 2000 m and $15^{\circ }$; (**b**) Ferry at 4000 m and $160^{\circ }$; (**c**) Frigate at 2000 m and $210^{\circ }$; (**d**) Passenger Ship at 4000 m and $332^{\circ }$; (**e**) Yard Ship at 4000 m and $332^{\circ }$.

Figure 14 shows examples of the model applied to synthetic images from the testing dataset. As theoretically predicted in the mAP analysis, it is possible to see that the model achieves high precision scores on the detection and classification even at greater distances and different bow angles the bow angles and distances at this image are not calculated but know from the dataset.

**Figure 15:**
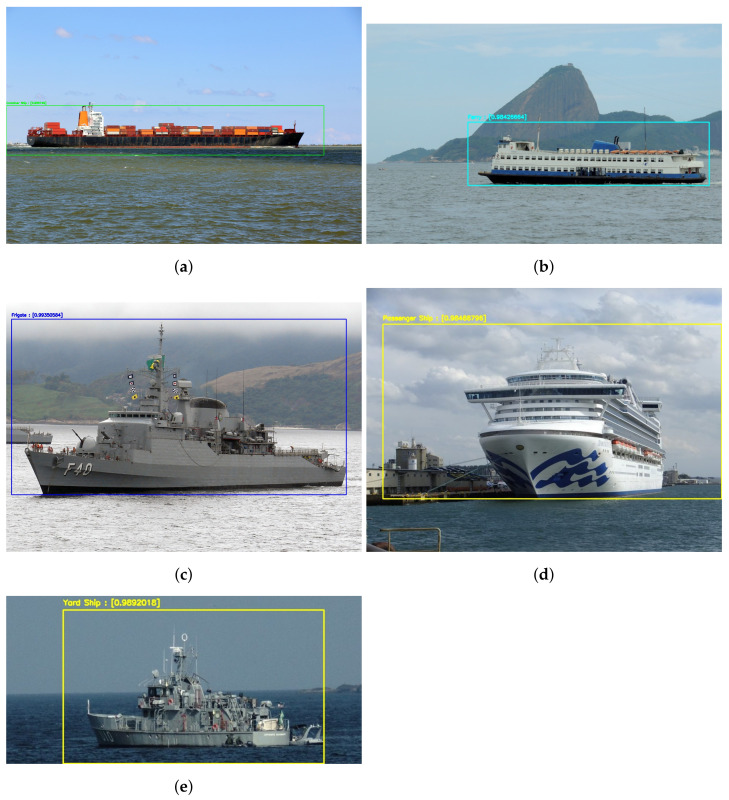
Results of the detection and classification model applied on real images from the Internet. The classification probabilities are: (**a**) Container Ship, 0.98; (**b**) Ferry, 0.98; (**c**) Frigate, 0.92; (**d**) Passenger Ship, 0.98; and (**e**) Yard Ship, 0.99.

We also tested our model on real images of the same kind of vessels and achieved good results, as can be seen in Figure 15. The vessels were detected in all the pictures, and the most likely class associated with each of the detections is to the correct class of ship. As can be seen in Figure 15, the model detected both Frigate and Yard Ships regardless of the similarities on both types of vessels.

#### 4.3.2. Field Testing

Finally, we tested our classification model using images captured from our XR periscope device. The probe uses a GoPro Fusion camera that captures video at up to 5.2 K/30 fps or 3 K/60 fps. Since it over-captures, it is possible to convert spherical content into traditional stills images and videos sequences. This camera has an advanced “gimbal-like” image stabilization system that prevents movement artifacts in the captured images.

**Figure 16:**
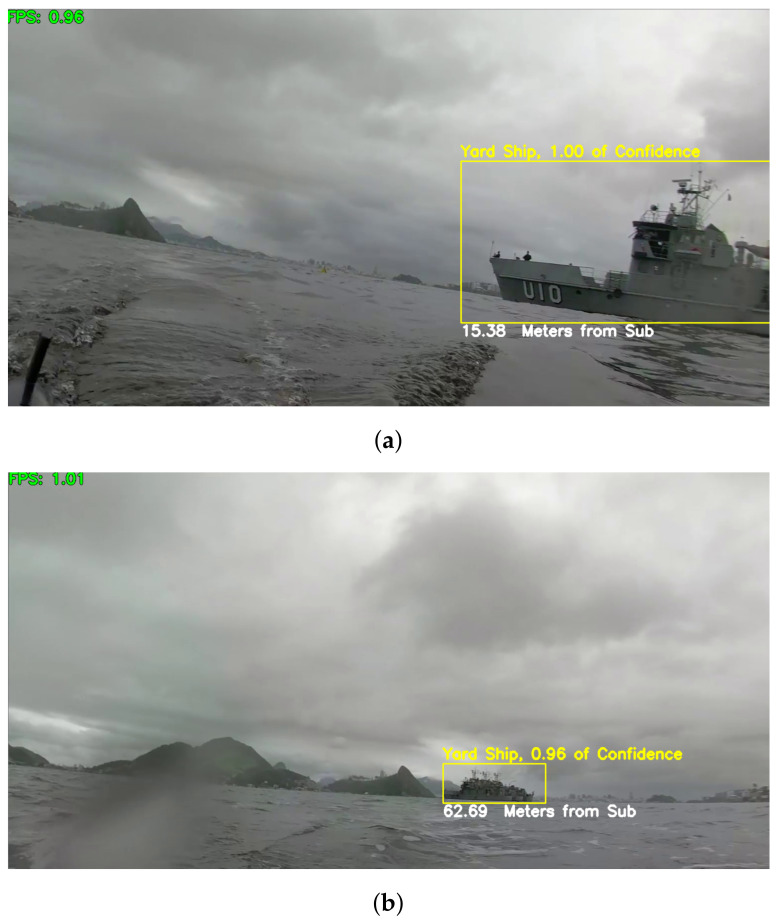
Results on two frames of a video sequence captured by our XR Periscope device. (**a**) Yard Ship at 15.38 m; (**b**) Yard Ship at 62.69 m.

We tested our solution, including software, and equipment with a Yard Ship of the Brazilian navy. Figure 16 illustrates the detection, classification, and distance estimation of the target ship in two frames of a video sequence. Note that results are consistent even under challenging weather conditions, with poor natural lighting due to a cloudy/rainy day and image distortions resulting from the camera lenses often get wet with saltwater.

It is possible to see a Yard Ship at 15.38 and 62.69 m, respectively. This test was performed using frames of a video captured by our probe

## 5. XR Periscope User Experience

Although the submarine community is not very large, with the help of the Brazilian navy, we were able to perform a simulation of the actual use of our XR Periscope. Then, we applied questionnaires to 19 experienced submarine officers.

The experiment consists of a submarine officer being told to perform a horizontal scan procedure with the XR Periscope. We formulated the following hypothesis to validate our proposal:

**Hypothesis** **1.** *The XR Periscope improves the security in the procedure to return to periscope depth.*

**Hypothesis** **2.** *The submarine tasks that involve observation of points and vessels of interest can be performed from the security quota with the XR Periscope.*

**Hypothesis** **3.** *The ship detection, classification, and distance estimation improve the navigation process.*

**Hypothesis** **4.** *The XR Periscope contributes to lower the general Submarine Discretion Fee (SDF).*

Since there is not yet a tactical and safety procedure defined by the Navy to use the equipment, we were not allowed to experiment using a real submarine. For this reason, we recorded a set of videos with the probe and used the $360^{\circ }$ videos with the VR device, simulating the environment of an actual periscope operation. We decided that for this test, the duration of the video should be 45 s, as the similar maneuver with the optical periscope has to be 30 s according to the perisher technique. Once the image recognition procedure was performed, 90 sequential frames were saved to be used experimentally with the virtual reality glasses. We used the HMD HTC Vive Pro, due to its facility for connecting with 360 video streaming and its high quality of display, resulting in a 2 fps rate.

We used the Unity platform [21] to visualize and manage these images in virtual reality. To give the freedom of the head movement, we developed a simple scenario composed of a sphere and the 360${}^{\circ }$ video projected on it through a skybox.

The relative direction of the user’s head to the vessel’s front is calculated and displayed in the simulation along with its heading and speed at each instant. To perform this calculation, we used the camera’s Y position about a fixed offset determined by the photo that indicates the position of the vessel’s front to the zero point of rotation of the photo image. This value can be recalculated with the variation of the images and undergo minor changes. The (simulated) submarine heading and speed are also displayed.

In order to reproduce a real situation, we created a fictional scenario, using the most likeable procedures of a submariner as possible. All participants have extensive experience with periscope operation. Each officer was told as the simulation began that:

*The submarine is in a fictitious location. The commander informed the periscope officer that he should perform a horizon scan from a security quota. The commander also informs the periscope officer that he must use a new system in the final stages of development by CASNAV and UFF University, the XR Periscope. The procedure developed by COMFORS indicates that the system must be hoisted to the surface from a depth of 42 m, as in Figure 2, for 45 s with a maximum speed of 5 knots.*

**Figure 17:**
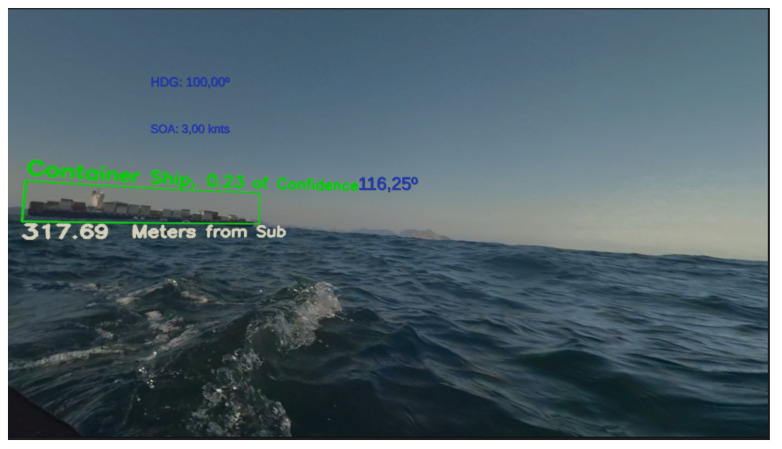
User Interface of the XR Periscope.

Figure 17 shows the user visualization on the XR Periscope with the heading, speed, and relative bearing information in blue. The recognized objects are also highlighted along with their distance estimation.

After using the X Periscope, the officer responds to a survey on his experience according to a Likert scale [22]. Question 1 asks the user’s experience level with the horizon scan procedure and Question 2 the experience with virtual reality. As can be seen in Table 3, the users have a good experience with the optical periscope horizon scan and a median level of experience with virtual reality.

Questions 3, 4, and 6 are related to our Hypothesis 1 and 2:

- Question 3: Does the XR Periscope helps in compiling the tactical scenario?
- Question 4: Assuming the XR Periscope can be launched from the security quota, could it help increase security in returning to the periscope quota?
- Question 6: Could the XR Periscope be used to perform secondary tasks?

As can be seen in Table 4, for Question 4, 93.75% of the users strongly agree and 6.25% agree that the XR Periscope would be helpful to increase the security in the procedure to return to periscope depth. This result agrees with Question 3, not rejecting Hypothesis 1. In the same table, in Question 6, 100% of the users strongly agree that the XR Periscope can be used to perform secondary tasks. Secondary tasks are the ones described in Hypothesis 2, not rejecting it either.

Question 8 asks: “Did the classification and distance of the contacts provided by the XR Periscope help compile the tactical scenarios ?”. It is a direct answer to Hypothesis 3, and as can be seen in Table 5, 87.5% of the users strongly agree and 12.5% of the users agree, not rejecting this hypothesis.

In Question 7, 93.75% of the users strongly agree and 6.25% agree that the XR Periscope increases navigation security.

Question 9 asks if the information is clearly displayed. Since 87.5% of the users strongly agree and 12.5% agree with this question, we assume that the interface is user-friendly. At last, Question 5 asks if the use of the XR Periscope helped in the decision-making process in the exercise, and 75.0% of the users strongly agree and 6.25% agrees with that question, showing that the equipment can be useful in the decision-making process. The results for Questions 5 and 9 can be seen in Table 6.

## 6. Conclusions and Future Work

Although we present the complete solution for a real submarine operation, our work still lacks tests in a real submarine at sea. Those tests would be extremely costly due to the harsh environment and high pressures that the submarine operates in. For doing so in the near future, an operation procedure of the equipment must be studied and validated by the submarine force.

Our results confirmed that the dataset of synthetic images is a viable alternative to performing object detection and classification in this specific scenario and from this point of view. Additionally, this type of dataset was shown to be less costly and faster to produce, more accessible to manipulate and label than real images.

The DAQ functions provide us with great possibilities to improve the trained detection and classification model since it allows training the CNN using both synthetic and real data. The DAQ automatizes collecting and labeling data in different real-life conditions that otherwise would be very difficult to manage.

Even though the existence of possible bias on the user experience tests due to the low frame rate and by not being able to test in the real environment, the achieved results confirm that the XR Periscope solution can be very useful for improving the safety of a submarine conduction, considerably increasing its operational efficiency by reducing the submarine discretion fee. It can also bring a profound revision of the “perisher” technique by using disruptive technology as it helps the commander decision process.

Our technique can be adapted and used in other vessels and cases, like port entrances, monitoring points of interest at sea, or as part of the control system of autonomous vessels. The rendering of other vessel classes can be easily implemented and included in the synthetic images dataset to increase the detection and classification model spectrum.

Besides that, other functionalities can be quickly developed, such as estimating the closest approach point, bow angle, and GODEX [23], which is the maximum period of time that the submarine can stay at periscope depth without risk of collision with other vessels. Those functionalities depend on a series of parameters such as ship speed and direction, other vessels’ directions and draught, and adaptation to different conditions like night vision, infrared images, and so forth.

## Figures and Tables

**Table 1:** We consider five classes of objects (Ship Type), the presence or the absence of background (Bg), and different distances of the object to the camera while generating synthetic images.

Distance (in Meters)											
Ship Type	Bg	500	1000	2000	3000	4000	5000	6000	7000	8000	10,000
Container Ship	Yes		✓	✓		✓		✓		✓	
	No		✓	✓		✓		✓		✓	✓
Ferry	Yes	✓	✓	✓	✓						
	No	✓	✓	✓	✓	✓		✓		✓	
Frigate	Yes	✓	✓	✓	✓						
	No	✓	✓	✓	✓	✓		✓		✓	
Passenger Ship	Yes		✓	✓		✓		✓			
	No		✓	✓		✓		✓	✓	✓	✓
Yard Ship	Yes	✓	✓	✓	✓						
	No	✓	✓	✓	✓	✓	✓	✓			

**Table 2:** Results of Mean Average Precision (mAP).

Ship Type	mAP	True Positive	False Positive
Container Ship	99.96%	4877	4
Ferry	99.99%	4973	2
Frigate	99.99%	4988	8
Passenger Ship	100.00%	255	0
Yard Ship	99.98%	4877	10

**Table 3:** Q1,Q2—User experience.

Question	Strongly Disagree	Disagree	Neutral	Agree	Strongly Agrees
Q1	0.00%	0.00%	25.00%	12.50%	62.50%
Q2	0.00%	12.50%	31.25%	25.00%	31.25%

**Table 4:** Q3, Q4, Q6—Hypothesis 1 and 2.

Question	Strongly Disagree	Disagree	Neutral	Agree	Strongly Agrees
Q3	0.00%	0.00%	0.00%	6.25%	93.75%
Q4	0.00%	12.50%	0.00%	6.25%	93.75%
Q6	0.00%	12.50%	0.00%	0.00%	100.00%

**Table 5:** Q7, Q8—Hypothesis 3.

Question	Strongly Disagree	Disagree	Neutral	Agree	Strongly Agrees
Q7	0.00%	0.00%	0.00%	6.25%	93.75%
Q8	0.00%	0.00%	0.00%	12.50%	87.50%

**Table 6:** Q5, Q9—User Interface.

Question	Strongly Disagree	Disagree	Neutral	Agree	Strongly Agrees
Q5	6.25%	0.00%	12.50%	6.25%	75.00%
Q9	0.00%	0.00%	0.00%	12.50%	87.50%

## Data Availability

Our dataset is publicly available at https://drive.google.com/drive/folders/1mI2BXuCd6n9ZmUqbNzCgyv8Zv9V_NLNn?usp=sharing.
